# Usefulness of Lateral Arm Free Flap in Heel Reconstructions After Malignant Skin Tumor Excision: An Observational Study

**DOI:** 10.3390/jcm15010192

**Published:** 2025-12-26

**Authors:** Soyeon Jung, Sodam Yi, Seokchan Eun

**Affiliations:** 1 Department of Plastic and Reconstructive Surgery, Hallym University College of Medicine, Hallym University Dongtan Sacred Heart Hospital, Hwaseong 18450, Republic of Korea; ps.soyeon.jung@gmail.com; 2 Department of Plastic and Reconstructive Surgery, Seoul National University College of Medicine, Seoul National University Bundang Hospital, Seongnam 13620, Republic of Korea; isds4774@snubh.org

**Keywords:** heel, oncologic defect, free flap, lateral arm flap

## Abstract

**Background/Objectives:** Heel reconstruction is a complex procedure that requires soft tissue reconstruction resistant to weight, pressure, and shear stress. Various flap reconstruction methods have been reported; among them, free fasciocutaneous flaps have advantages in terms of function and aesthetics, but also have challenges due to the longer operation time required and the possibility of failure. The primary aim of this study was to examine the functional outcomes of heel reconstruction using free lateral arm fasciocutaneous flaps after wide excision of heel skin cancer. **Methods:** Between January 2014 and December 2020, eight patients underwent wide excision of skin cancer and reconstruction of the heel with a lateral arm free flap. Perioperative clinical data and postoperative outcomes, including flap survival, complications, Lower Extremity Functional Scale (LEFS) score, and American Orthopaedic Foot and Ankle Society scale (AOFAS) score, were analyzed from clinical records. Functional assessments were performed at a minimum of 12 months postoperatively (mean 18.3 months, range 12–24 months) by a single blinded examiner who was not involved in the surgical procedures. Both preoperative and postoperative LEFS and AOFAS scores were recorded for comparison. **Results:** The mean size of the skin and soft tissue defect was 32 cm^2^, the mean duration of surgery was 179 (range: 160–215) minutes, and the mean duration of hospital stay after surgery was 17 (range: 14–19) days, with a mean follow-up period of 48 (range: 33–59) months. Among the eight patients, two had diabetes mellitus (25%), one had peripheral neuropathy (12.5%), and none had clinically significant peripheral vasculopathy. All flaps survived, with one congestive episode. Satisfactory aesthetic and functional results were observed in all patients. The mean preoperative LEFS score was 28 (SD ± 6.1), which improved significantly to a postoperative mean of 57 (SD ± 8.3). Similarly, the mean preoperative AOFAS score was 45 (SD ± 5.8), improving to a postoperative mean of 61 (SD ± 6.2). Minor donor site complications included hypertrophic scarring in two patients (25%) and transient sensory changes in the lateral arm region in three patients (38%), all of which resolved with conservative management. **Conclusions:** This research suggests that the lateral arm free flap can be considered a reliable option in heel reconstruction, resulting in acceptable functional and aesthetic outcomes. It provides excellent durability, with solid bony union and good contour in small to moderate-sized heel defect cases.

## 1. Introduction

The heel has particular histologic characteristics, namely, a thick epidermis and dermis with very thin subcutaneous tissue consisting of numerous fibrous septa traversing the skin to the plantar aponeurosis [1]. These septa divide the subcutaneous tissue into small loculi that act as an impact-absorbing cushion and prevent the skin from sliding. The adherence between the dermis and the underlying muscle allows the entire weight of the body to be supported while standing [2]. Defects of the heel arise most commonly from trauma, followed by tumor extirpation, chronic wounds, burns, and ulcers. In particular, skin cancer that occurs on the heel is often of the melanoma type and requires the excision of a wide area, resulting in a large defect.

Surgeons agree that the main goal of heel reconstruction is to provide durable coverage that allows patients to walk properly, have a normal appearance, and yield acceptable donor site morbidity [3,4]. The loss of the shock-absorbing and friction-resistant heel tissue leads to changes in gait patterns. However, soft tissue defects of the heel represent a challenging problem due to the low mobility of adjacent tissues, limited vascularity, and lack of units available for wound reconstruction [5,6]. Flap donor selection is particularly difficult due to its anatomic complexity and unique role in the walking motion. Basically, flap coverage is preferred to enhance the restoration of the weight-bearing. Thus, diverse local flaps or free flaps are considered primarily to reconstruct the heel area [7,8,9,10,11,12,13,14,15,16,17,18].

The aim of the present study is to share our experience and outcomes in the surgical reconstruction of oncologic heel defects using a lateral arm free flap. A lateral arm flap is a reliable option with a consistent vascular anatomy to reconstruct the various types of defects. Thus, the flap can be utilized for the heel defect following skin cancer ablation. However, very few cases of heel reconstruction with lateral arm free flap following tumor resection have been reported. Our study provides useful information regarding surgical results and patient satisfaction with validated functional scales.

## 2. Materials and Methods

The present study included eight patients who underwent wide excision of heel skin cancer and soft tissue reconstruction with lateral arm free flaps between 2014 and 2020.

All patients provided written informed consent before inclusion in the study. The study was performed in accordance with the Declaration of Helsinki and approved by the Institutional Review Board of Seoul National University Bundang Hospital (B-2503-959-102).

Patient selection and criteria:

Inclusion criteria: (1) pathologically confirmed malignant skin tumor of the heel requiring wide excision, (2) defect size amenable to lateral arm flap coverage (estimated 20–60 cm^2^), (3) adequate recipient vessels (posterior tibial artery and veins) confirmed by preoperative CT angiography, and (4) absence of severe peripheral vascular disease (defined as ankle–brachial index < 0.6).

Exclusion criteria: (1) patients with inadequate follow-up duration (<12 months), (2) prior ipsilateral upper extremity surgery or trauma that would preclude flap harvest, (3) patients who declined free flap reconstruction and opted for alternative treatment, and (4) active infection at the heel defect site requiring staged reconstruction.

Data regarding the demographic characteristics of the patients, including age, sex, tumor type and staging (according to AJCC 8th edition), presence of comorbidities (diabetes mellitus, peripheral vasculopathy, neuropathy), defect size, pedicle vessels, duration of surgery, time of immobilization during postoperative care, adjuvant treatment (lymph node dissection, immunotherapy), follow-up period, patient satisfaction, and surgical outcomes, were collected retrospectively with medical records. We also gathered data regarding free flap complications such as episodes of congestion or arterial insufficiency, flap failure, and emergency reoperations. Long-term functional results were evaluated based on the Lower Extremity Functional Scale (LEFS) and American Orthopaedic Foot and Ankle Society scale (AOFAS) scores, which were obtained from clinical records. Donor site complications, including hypertrophic scar formation, sensory changes, and functional impairment, were systematically documented (Table 1).

### 2.1. Surgical Technique

The entire upper extremity up to the axilla was prepared and draped, with the patient in a supine position. The complete removal of malignant tumors of the heel was carried out with an adequate surgical margin. The ipsilateral shoulder was elevated and rested on a pad, with the elbow in a flexed position. The lateral arm flap was outlined with a surgical skin marker. The borders of the lateral arm flap included the deltoid tuberosity superiorly and the lateral epicondyle of the elbow inferiorly. The proposed flap was centered over the lateral intermuscular septum, which courses from the deltoid insertion to the lateral epicondyle. First, the posterior flap was elevated deep to the muscular fascia over the triceps, which was peeled anteriorly until the septum was encountered. This fascia was included in the flap to preserve vascularity. As the flap was elevated toward the septum, small muscular perforators were encountered and had to be coagulated or ligated. The pedicle and septum were identified together and exposed from distal to proximal. Then, the anterior aspect of the flap was elevated. The same dissection was performed to expose the brachialis and brachioradialis muscles. The fascia here was slightly more attached to the muscles. The artery and vein at the distal aspect were ligated and divided. Staying deep to these vessels, the intermuscular septum was released to the level of the humeral periosteum, and the septum was then raised, with the periosteum, from the humerus proximally to the level of the deltoid insertion. The dissection continued along the septum, and care was taken to protect the radial nerve. The anterior radial collateral artery was ligated proximally. Once an adequate pedicle length was obtained, the PRCA and venae comitantes were divided and used as the pedicle of the lateral arm flap. If the neurosensory flap was to be harvested, the posterior cutaneous nerve of the arm, which enters the skin flap proximally and superficial to the triceps fascia, was identified and divided proximally. This flap’s pedicles were used in either an end-to-end or end-to-side fashion. After flap transfer and micro-anastomoses, the donor site wound was closed primarily.

### 2.2. Tips and Tricks

1. Flap design and markings: The lateral intermuscular septum is reliably located by drawing a line from the deltoid insertion to the lateral epicondyle. The skin paddle is centered over this line. Doppler ultrasound can be used preoperatively to confirm the septocutaneous perforators, typically located 2–4 cm proximal to the lateral epicondyle.
2. Dissection sequence: We recommend starting dissection from the posterior border to identify the lateral intermuscular septum early. This “septum-first” approach provides a clear anatomical landmark and reduces the risk of inadvertent injury to the pedicle. The posterior cutaneous nerve of the arm can be harvested with the flap if sensory restoration is desired.
3. Radial nerve protection: The radial nerve runs along the lateral intermuscular septum and is at risk during pedicle dissection. Gentle retraction and careful dissection under loupe magnification (at least 2.5×) are essential. The nerve typically lies deep to the pedicle at the level of the mid-humerus.
4. Pedicle length: To obtain adequate pedicle length (typically 6–8 cm), the dissection should be carried proximally to the level of the deltoid insertion. The posterior radial collateral artery branches from the profunda brachii artery in this region, providing a reliable pedicle with sufficient caliber (artery diameter 1.5–2.5 mm, vein diameter 2.0–3.5 mm) for microvascular anastomosis.
5. Donor site closure: Primary closure is feasible for a donor site less than 7 cm. For wider flaps, we perform limited undermining of the surrounding skin to facilitate tension-free closure, avoiding skin grafting when possible to minimize donor site morbidity.

### 2.3. Functional Outcome Analysis

The primary outcomes included overall flap survival and postoperative ambulatory status. Long-term functional results were evaluated based on the Lower Extremity Functional Scale (LEFS) and American Orthopaedic Foot and Ankle Society scale (AOFAS) scores. Preoperative baseline scores were obtained within 2 weeks before surgery. Postoperative functional assessments were conducted at a minimum of 12 months after surgery (mean 18 months, range 12–24 months) by a single blinded examiner: a physical therapist who was not involved in the surgical procedures and was unaware of intraoperative details. This blinded assessment approach minimized evaluation bias and ensured objective functional outcome measurement (Table 1).

## 3. Results

The present study included a total of eight patients—five males and three females—with a mean age of 58 (range: 42–83) years. The mean size of the skin and soft tissue defect was 32 cm^2^. All patients underwent wide excision of malignant heel tumors and reconstruction with lateral arm free flaps. The mean duration of surgery was 179 (range: 160–215) minutes. Routine laboratory investigations, including a complete blood count, admission panel, and coagulation panel, were performed until 3 days after surgery. The mean duration of hospital stay after surgery was 16.8 (range: 14–19) days, with a mean follow-up period of 49 (range: 33–59) months.

Among the eight patients, comorbidities potentially affecting surgical outcomes included diabetes mellitus in two patients (25%, both type 2 with HbA1c < 7.5%), peripheral neuropathy in one patient (13%, secondary to diabetes), and no clinically significant peripheral vasculopathy (all patients had ankle-brachial index > 0.9). Tumor types included six melanomas and two squamous cell carcinomas. Melanoma staging according to the AJCC 8th edition included: three pT1N0M0, two pT2N0M0, and one pT1N1M0. The two squamous cell carcinomas were pT1N0M0 and pT2N0M0. Three patients underwent sentinel lymph node biopsy (38%), with one patient showing positive inguinal lymph node involvement requiring complete lymph node dissection. Two patients with advanced melanoma received adjuvant immunotherapy (pembrolizumab) postoperatively (25%).

There was one episode of flap congestion, which was spontaneously resolved without emergency or secondary operation. Donor site complications included hypertrophic scarring in two patients (25%), which was managed conservatively with silicone gel sheeting and pressure garments with satisfactory flattening within 6–9 months. Three patients (38%) experienced transient sensory changes in the lateral arm and forearm region, characterized by mild hypoesthesia, which gradually improved over 12–18 months. No patient required secondary surgery for donor site complications. All donor sites were closed primarily without the need for skin grafting.

During the long-term follow-up period, all patients survived the first 12 months, but one patient later expired due to inguinal lymph node and lung metastasis. All lateral arm flaps remained intact without ulcer formation and provided a deep level of protective sensation sufficient for everyday use. Although formal sensory testing with Semmes-Weinstein monofilaments was not performed in this retrospective series, protective sensation was clinically assessed by the ability to perceive light touch with a cotton swab and sharp/dull discrimination, which all patients demonstrated at final follow-up. The maintenance of protective sensation is attributed to gradual reinnervation from the surrounding native heel tissue at the flap margins, a process well-documented in non-neurosensory free flaps used for weight-bearing reconstruction [19,20]. Most patients were satisfied with the overall appearance of the foot and heel contour.

The mean preoperative LEFS score was 28 (SD ± 6.1), reflecting significant functional impairment due to the tumor burden and associated pain. Postoperatively, the mean LEFS score improved significantly to 57 (SD ± 8.3), indicating substantial functional recovery. Similarly, the mean preoperative AOFAS score was 45 (SD ± 5.8), improving to a postoperative mean of 61 (SD ± 6.2). These improvements represent clinically meaningful gains, as prior studies suggest that LEFS improvements of >9 points and AOFAS improvements of >10 points exceed the minimal clinically important difference (MCID) for lower extremity reconstruction [21,22] (Table 1).

### 3.1. Case Description

#### 3.1.1. Case 1

**Figure 1:**
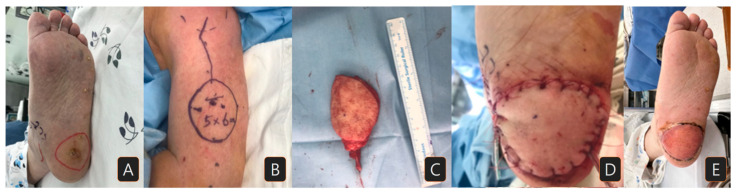
A case of a patient with squamous cell carcinoma on his left heel: (**A**) preoperative surgical margin of wide excision; (**B**) lateral arm free flap design (5 × 6 cm size); (**C**) elevated flap; (**D**) immediately after flap insertion; (**E**) three months after flap reconstruction.

An 83-year-old male presented to our clinic with a skin lesion of increasing size located on the left heel. Biopsy proved the lesion to be a squamous cell carcinoma, staged as pT2N0M0. The patient had no significant medical comorbidities (Figure 1A). Preoperative examinations showed no sign of distant metastasis. After removing the lesion with a 1.5 cm margin, a 5 × 6 cm sized lateral arm flap was elevated from the left upper arm (Figure 1B,C). The radial collateral artery of the profunda brachii artery and its venae comitantes were anastomosed to the right posterior tibial artery and vein. The donor site was primarily closed. At 29 months of follow-up, the patient exhibited no impairments in gait and did not experience any complications (Figure 1D,E).

#### 3.1.2. Case 2

**Figure 2:**
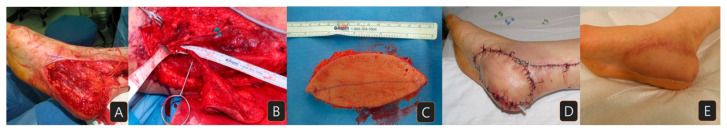
A case of a patient with melanoma on her right heel: (**A**) heel defect after wide excision of the cancer; (**B**) the elevated flap with the pedicle; (**C**) the harvested flap (5 × 10 cm); (**D**) immediate view after flap reconstruction; (**E**) eight-month postoperative view.

A 52-year-old female patient presented with a malignant melanoma measuring 2 × 4.5 cm on her right heel, staged as pT3N0M0 (Breslow thickness 3.2 mm). She had no relevant medical history. The lesion was excised with a 2 cm margin, resulting in a heel defect measuring 6 × 10 cm (Figure 2A). A lateral arm flap was elevated with the radial collateral artery of the profunda brachii artery and its venae comitans (Figure 2B,C). After flap transfer, micro-anastomoses were carried out between the flap pedicle and posterior tibial artery and vein in an end-to-side and end-to-end manner. The flap survived well with no event (Figure 2D). Eighteen months after the surgery, the patient was satisfied with the appearance of the flap and had no problem when walking or wearing shoes (Figure 2E).

#### 3.1.3. Case 3

**Figure 3:**
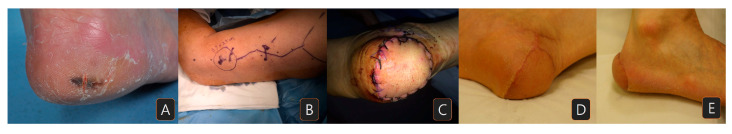
A case of melanoma on the right posterior heel: (**A**) preoperative view; (**B**) skin marking for flap elevation; (**C**) immediately after lateral arm flap reconstruction (4 × 5 cm in size); (**D**,**E**) five-month postoperative view.

A 79-year-old man with no underlying disease presented at the clinic with an irregular margin nevus with dimensions of 1 × 2.5 cm on his right heel (Figure 3A). The nevus rapidly increased in size over a short period, and the punch biopsy result was malignant melanoma. Preoperative MRI, chest and abdomen CT images, and positron emission tomography scans showed no signs of regional or distant metastasis. The lesion was excised with a 2 cm margin, resulting in a right heel defect with dimensions of 4 × 5 cm, with exposure of the calcaneal bone. A lateral arm flap was elevated from his right arm, and the radial collateral artery and its venae comitans were anastomosed to the right posterior tibial artery (Figure 3B). The flap settled well, and the coverage was successful (Figure 3C). At 12 months of follow-up, the patient had no impairments in walking and was satisfied with the appearance (Figure 3D,E), but lymph node metastases were newly found. Despite the adjuvant immunotherapy with pembrolizumab, he expired from eventual lung metastasis after 33 months of observation.

## 4. Discussion

The heel is unique in structure and well-adapted to the task of providing a weight-bearing surface and shock absorption when walking. It is estimated that the heel bears between 50 and 80% of the body weight when standing. For this function, the heel consists of a thick epidermis, and the epidermal–dermal complex is firmly anchored to the plantar aponeurosis by perpendicular fibrous septa [2,3]. These septa anchor the skin to the bone to prevent shearing. Between these vertical septa lie loculi of fat, which act as a shock-absorbing cushion for the heel [2,4]. An ideal technique for heel reconstruction should provide a durable and comfortable weight-bearing surface, solid anchoring to deep tissue for resistance to shear forces, and adequate protective sensation. Therefore, the reconstruction of these particular defects poses one of the most significant challenges after surgical resection [5,6]. Despite the considerable variety of flaps, the selection of the most suitable for the reconstruction of the heel remains debatable.

Pedicled fasciocutaneous flaps, such as the medial plantar flap, can provide a thin, pliable tissue that is easily contoured [12]. Although this method may be a good choice for most cases of heel defects, it has some limitations for long linear defects or large defect areas [13]. Besides, another limitation from the pedicle length needs to be considered [9]. Reconstruction of the foot using free flaps has been widely performed for the last two decades. The scapular flap is often bulky and incapable of innervation, and donor site morbidity and hypertrophic scar formation may be conspicuous in deltoid flaps [14]. In dorsalis pedis flaps, dissection is difficult, the amount of soft tissue is limited, and there is significant potential donor site morbidity [15]. Groin flaps have a short pedicle, with variable arterial and venous anatomy [16]. Radial forearm flaps may be associated with unsightly donor site distortion or skin grafting, and possible hair growth on the flap [17]. The anterolateral thigh perforator flap provides a large, pliable skin island with sufficient bulk for complex defect coverage. It can later be tailored via debulking to provide thin coverage, allowing contouring of the surface. The anterolateral thigh flap contains thick femoral fascia that can withstand pressure and shearing force, making it a good candidate for use in heel reconstruction [18,19]. According to the literature, the anterolateral thigh flap is the most common flap to reconstruct the heel [11]. It can also incorporate the muscle component depending on the need for heel padding [11]. Lastly, skin grafting can be one of the options to treat the skin defect of the heel. However, it is not reliable for durability or weight-bearing owing to the nature of the skin. Only when treating the heel defect from an avulsive injury can the detached skin be used to graft back to the area [20].

The lateral arm flap, first described by Song et al., is seldom used to cover heel defects [21]. The lateral arm free flap has gained popularity, particularly for resurfacing small skin defects on the hands and upper limbs [22]. This flap can be raised quickly and easily, saving considerable operation time without requiring any positional changes during the operation. In this retrospective analysis, we showed that the free lateral arm flap meets the above criteria by providing stable healing and contour correction [23]. The thickness of a lateral arm flap is between that of a radial forearm and scapular flap. It is not bulky, yet consists of a dermis and subcutaneous tissue with sufficient thickness to provide stability, and it is pliable [24]. The pedicle is an end artery with sufficient length and diameter such that a major artery is not sacrificed, as in radial forearm flaps. Donor- and recipient-site surgeries can proceed simultaneously without the need for patient repositioning [23,24]. In one study, the dimensions of plantar defects in this series of lateral arm flaps ranged from relatively small to moderate. This might have allowed for neurotization from surrounding tissues, and with regular rehabilitation and protection, relatively higher shearing forces were tolerated [25,26].

Although melanoma and squamous cell carcinoma differ in their biologic behavior and recommended resection margins (melanoma typically requiring 1–2 cm margins depending on Breslow thickness versus SCC requiring 0.5–1 cm margins for most cases), we grouped these tumor types together in this analysis for the following reasons: (1) Both tumor types occurred on the heel and required oncologically adequate wide excision resulting in full-thickness defects requiring free flap reconstruction. (2) The primary outcome of this study was the functional and aesthetic success of lateral arm free flap reconstruction, not oncologic outcomes, and the reconstructive principles and technical considerations were identical regardless of tumor type. (3) The resulting defect size range (20–60 cm^2^) overlapped considerably between melanoma and SCC cases in our series. (4) Both tumor types necessitate durable, weight-bearing soft tissue coverage, and the lateral arm flap’s biomechanical properties address these requirements equally. Future prospective studies with larger cohorts would enable subgroup analysis by tumor type to examine whether oncologic outcomes differ, but for the purpose of evaluating reconstructive technique feasibility and functional outcomes, this combined analysis is appropriate.

In our study, without flap reinnervation, most of the flaps still showed deep protective sensation sufficient for daily life. None of the patients developed ulceration after the surgery. Ulceration is one of the most commonly observed complications of free flap reconstruction of the heel. Although we did not perform formal sensory testing with Semmes-Weinstein monofilaments in this retrospective series, protective sensation was clinically evaluated by assessing the patient’s ability to perceive light touch with a cotton swab and to distinguish sharp from dull stimuli. All patients demonstrated these abilities at final follow-up, indicating the presence of protective sensation. The physiologic basis for this sensory recovery in non-neurosensory flaps is gradual reinnervation from adjacent native heel tissue at the flap margins, a well-documented phenomenon in weight-bearing foot reconstruction. Previous studies by Sönmez et al. and Potparic and Rajac have demonstrated that non-neurosensory free flaps can achieve protective sensation over time through peripheral reinnervation, with functional outcomes comparable to neurosensory flaps [25,26]. Our clinical experience aligns with these findings, supporting the use of lateral arm flaps without formal nerve coaptation for heel reconstruction.

Lastly, results regarding the dysfunction of walking, shoe-wearing, and appearance were obtained subjectively from the patients. This study describes the impact on ambulation, functional recovery, and patient satisfaction in patients who underwent plantar reconstruction using free flaps. Functional outcomes after free flap reconstruction in the heel area have not been extensively evaluated in previous studies. We analyzed the functional status for each patient, gathering data from clinical records using the Lower Extremity Functional Scale (LEFS) and the American Orthopaedic Foot and Ankle Society scale (AOFAS). The LEFS is a 20-item questionnaire, with each item graded on a Likert scale from 0 (indicating severe difficulty or inability to perform any activity) to 4 (indicating no difficulty). The maximum possible score is 80 points, indicating optimal function, while the minimum possible score is 0 points, indicating no function [27]. The subjective components of the LEFS and AOFAS are validated indicators of outcomes and evolution [28]. These scales have been used to evaluate pain and functional outcomes related to daily walking activities [29]. When evaluating specific data points post-surgery, we found that most patients were able to perform daily activities without deficits. We further believe that a lateral arm flap comprising less subcutaneous fatty tissue, with theoretically less shearing stress, might have contributed to the successful outcomes.

This study has several limitations that should be acknowledged. First, the sample size was small (*n* = 8), limiting the statistical power to detect differences in outcomes between subgroups (e.g., melanoma versus SCC, diabetic versus non-diabetic patients). Second, the retrospective design introduces potential selection bias and precludes standardized prospective data collection. Third, functional assessments relied on subjective scales (LEFS and AOFAS) without objective biomechanical gait analysis or quantitative sensory testing with Semmes–Weinstein monofilaments. Fourth, we did not perform formal flap reinnervation, and protective sensation recovery was assessed clinically rather than with validated sensory testing protocols. Fifth, the study lacked a comparison group (e.g., other free flap types such as the anterolateral thigh flap) to determine the relative advantages of the lateral arm flap. Despite these limitations, our findings provide valuable preliminary evidence supporting the lateral arm free flap as a viable option for heel reconstruction in carefully selected patients.

## 5. Conclusions

In challenging situations requiring heel pad reconstruction, various candidates for free flap reconstruction were considered to optimize outcomes. Satisfactory results with minimal donor site morbidity were achieved using lateral arm free flaps. These procedures were associated with improvement in functional scales, high rates of patient satisfaction, and the ability to use normal footwear without added surgical complications at the donor or reconstruction sites. This observational study suggests that the lateral arm free flap represents a suitable reconstructive option for small to moderate-sized oncologic heel defects, offering acceptable functional and aesthetic outcomes with manageable donor site morbidity.

## Figures and Tables

**Table 1:** Patient characteristics, surgical details, and functional outcomes after heel reconstruction.

No	Sex	Age	Cancer Types	Staging	Defect Size (cm^2^)	DM	Neuropathy	LN Dissection	Duration of Surgery (min)	Hospital Stay (days)	Follow-up (months)	Peri-Operative Complications	Preop LEFS	Postop LEFS	Preop AOFAS	Postop AOFAS
1	M	62	Melanoma	pT1N0M0	35	No	No	SLNB (negative)	180	18	44	None	32	58	48	65
2	F	46	Melanoma	pT2N0M0	42	No	No	SLNB (negative)	165	16	58	None	26	54	34	64
3	M	83	SCC	pT2N0M0	30	Yes	No	No	170	17	48	None	24	56	46	53
4	M	45	Melanoma	pT1N0M0	24	No	No	No	215	21	35	Flap congestion	28	54	54	67
5	F	52	Melanoma	pT2N0M0	50	No	No	SLNB (negative)	185	18	51	None	30	59	43	58
6	M	53	SCC	pT1N0M0	20	Yes	Yes	No	190	19	52	None	25	64	44	59
7	F	42	Melanoma	pT1N0M0	35	No	No	No	160	16	62	None	31	51	41	63
8	M	79	Melanoma	pT1N1M0	20	No	No	CLND	170	17	33	Death	31	56	47	62

Abbreviations: DM = diabetes mellitus; LN = lymph node; SLNB = sentinel lymph node biopsy; CLND = complete lymph node dissection; LEFS = Lower Extremity Functional Scale; AOFAS = American Orthopaedic Foot and Ankle Society Scale; SCC = squamous cell carcinoma.

## Data Availability

All datasets that the conclusion is based upon are included in the manuscript. The datasets analyzed during the current study are available from the corresponding author upon reasonable request.

## Abbreviations

The following abbreviations are used in this manuscript.

LEFS	Lower Extremity Functional Scale
AOFAS	American Orthopaedic Foot and Ankle Society Scale
AJCC	American Joint Committee on Cancer
SCC	Squamous Cell Carcinoma
SLNB	Sentinel Lymph Node Biopsy
CLND	Complete Lymph Node Dissection
DM	Diabetes Mellitus
MCID	Minimal Clinically Important Difference
